# The Fumarate Reductase of *Bacteroides thetaiotaomicron*, unlike That of *Escherichia coli*, Is Configured so that It Does Not Generate Reactive Oxygen Species

**DOI:** 10.1128/mBio.01873-16

**Published:** 2017-01-03

**Authors:** Zheng Lu, James A. Imlay

**Affiliations:** Department of Microbiology, University of Illinois, Urbana, Illinois, USA; University of Michigan

## Abstract

The impact of oxidative stress upon organismal fitness is most apparent in the phenomenon of obligate anaerobiosis. The root cause may be multifaceted, but the intracellular generation of reactive oxygen species (ROS) likely plays a key role. ROS are formed when redox enzymes accidentally transfer electrons to oxygen rather than to their physiological substrates. In this study, we confirm that the predominant intestinal anaerobe *Bacteroides thetaiotaomicron* generates intracellular ROS at a very high rate when it is aerated. Fumarate reductase (Frd) is a prominent enzyme in the anaerobic metabolism of many bacteria, including *B. thetaiotaomicron*, and prior studies of *Escherichia coli* Frd showed that the enzyme is unusually prone to ROS generation. Surprisingly, in this study biochemical analysis demonstrated that the *B. thetaiotaomicron* Frd does not react with oxygen at all: neither superoxide nor hydrogen peroxide is formed. Subunit-swapping experiments indicated that this difference does not derive from the flavoprotein subunit at which ROS normally arise. Experiments with the related enzyme succinate dehydrogenase discouraged the hypothesis that heme moieties are responsible. Thus, resistance to oxidation may reflect a shift of electron density away from the flavin moiety toward the iron-sulfur clusters. This study shows that the autoxidizability of a redox enzyme can be suppressed by subtle modifications that do not compromise its physiological function. One implication is that selective pressures might enhance the oxygen tolerance of an organism by manipulating the electronic properties of its redox enzymes so they do not generate ROS.

## IMPORTANCE

Whether in sediments or pathogenic biofilms, the structures of microbial communities are configured around the sensitivities of their members to oxygen. Oxygen triggers the intracellular formation of reactive oxygen species (ROS), and the sensitivity of a microbe to oxygen likely depends upon the rates at which ROS are formed inside it. This study supports that idea, as an obligate anaerobe was confirmed to generate ROS very rapidly upon aeration. However, the suspected source of the ROS was disproven, as the fumarate reductase of the anaerobe did not display the high oxidation rate of its *E. coli* homologue. Evidently, adjustments in its electronic structure can suppress the tendency of an enzyme to generate ROS. Importantly, this outcome suggests that evolutionary pressure may succeed in modifying redox enzymes and thereby diminishing the stress that an organism experiences in oxic environments. The actual source of ROS in the anaerobe remains to be discovered.

## INTRODUCTION

The oxygenation of the planet occurred late in evolutionary time (1), and it imposed a crisis upon extant microbes. Molecular oxygen is toxic. It can directly poison specialized free radical and low-potential enzymes that are found in some anaerobes (2, 3). More generally, oxygen also can intercept some of the electrons that flow through redox enzymes, thereby generating superoxide and hydrogen peroxide (4). These species are stronger oxidants than is oxygen itself, and they rapidly oxidize the exposed iron cofactors on families of [4Fe-4S] dehydratases (5–8) and mononuclear iron enzymes (9–11). The oxidized iron atoms dissociate from those enzymes, activities are lost, and their pathways stop working. The outcome is a cessation of metabolism and growth.

Such oxidant-sensitive enzymes are almost universally distributed through the biota, and so aerobic organisms have invented ways to protect them. The primary defense is the synthesis of superoxide dismutases (SOD) that scavenge O_2_^−^ and of peroxidases and catalases that scavenge H_2_O_2_. In the model bacterium *Escherichia coli*, these enzymes are among the most abundant in the cell, yet their collective activity is barely sufficient to keep reactive oxygen species (ROS) concentrations below the threshold for overt toxicity (12). Mutant strains that lack either SOD or catalase/peroxidase activities are unable to grow in a simple glucose medium (13, 14).

Obligate anaerobiosis represents a scenario in which evolution has failed to equip microbes for oxygen exposure. The reason that oxygen poisons anaerobic metabolism is an issue of great interest to investigators of microbial ecology, pathogenesis, and industrial fermentations. Experimental evidence suggests that endogenous ROS production may be a key element. *Bacteroides thetaiotaomicron* is a dominant obligate anaerobe in the human intestine (15), and it provides an apt contrast to *E. coli*. Both are intestinal microbes that catabolize carbohydrates found in that environment; however, whereas *E. coli* thrives upon excretion into oxic surface waters, *Bacteroides* becomes quiescent. Metabolic analysis indicates that oxygenation inactivates two key enzymes in the *B. thetaiotaomicron* central metabolism: pyruvate:ferredoxin oxidoreductase (POR), which may be directly damaged by oxygen itself, and fumarase (16). The latter enzyme belongs to the iron-sulfur dehydratase family that is especially vulnerable to O_2_^−^ and H_2_O_2_. In *B. thetaiotaomicron*, these enzymes remain inactive for the duration of aeration; when anoxia is restored, the enzymes are reactivated, and growth resumes.

In striking contrast, the *E. coli* fumarase enzymes maintain full activity upon aeration. This discrepancy is unlikely to derive from a difference in the titers of scavenging enzymes in the two organisms. *B. thetaiotaomicron* exhibits SOD activity that is similar to that of *E. coli*, and it has four distinct enzymes that can degrade H_2_O_2_ (17). Instead, an attractive possibility is that these organisms differ in the rates at which ROS are formed inside the aerated cell. If ROS are formed especially rapidly in *B. thetaiotaomicron*, then standard levels of scavenging enzymes may be insufficient to protect ROS-sensitive enzymes.

Our understanding of the mechanism of ROS production has lagged behind our knowledge of ROS-mediated damage. *In vitro* studies have identified quite a few enzymes that release ROS as inadvertent by-products when they operate in oxic solutions (4, 18, 19). In each case, they are flavin-dependent redox enzymes, and O_2_^−^ and H_2_O_2_ are formed when oxygen collides adventitiously with their flavins at the point in the catalytic cycle when the flavin is reduced. In the adventitious reactions, molecular oxygen competes with the physiological acceptor for the reduced enzyme.

**FIG 1:**
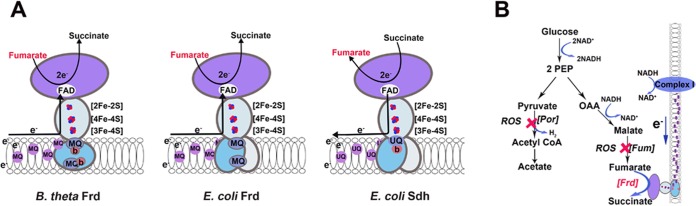
Physical structure and metabolic role of *B. thetaiotaomicron* fumarate reductase. (A) Structural diagrams of *B. thetaiotaomicron* fumarate reductase (Frd), *E. coli* Frd, and *E. coli* succinate dehydrogenase (Sdh). Physiological directions of electron flow are indicated by arrows. MQ, menaquinone; UQ, ubiquinone. Both cluster-proximal and -distal quinone binding sites were visualized in the *E. coli* Frd structure (36), and they are tentatively inferred for the *B. thetaiotaomicron* structure (66). (B) Role of Frd in redox balancing during the fermentation of glucose by *B. thetaiotaomicron*. Glycolysis generates 2 mol of NADH, which are recycled by malate dehydrogenase and by the Frd-dependent electron transport chain. Red crosses indicate the inactivation of pyruvate:ferredoxin oxidoreductase (Por) and fumarase (Fum) when the cell is aerated.

The rates at which different flavoenzymes leak electrons to oxygen vary widely (18), and it seems likely that the organisms that struggle the most with oxygen are those with the highest titers of the leakiest enzymes. To identify such enzymes, it is useful to pinpoint the physical traits that predispose flavoenzymes to react with oxygen. Comparative studies have been performed upon members of the complex II enzyme family. This family received particular attention because succinate dehydrogenase (Sdh) is a source of ROS within the mammalian respiratory chain (20–24). Sdh is a respiratory enzyme that transfers electrons from succinate to the quinone pool (Fig. 1A); autoxidation occurs when oxygen intercepts electrons from its reduced flavin (24, 25). The Sdh family also includes aspartate oxidase and fumarate reductase, both of which generate ROS markedly more quickly than does Sdh itself (25). Analysis of these enzymes revealed that ROS production is maximized when the flavin is highly solvent exposed, when it has a low reduction potential, and when it is the center of electron density on the reduced enzyme. These studies also revealed that aspartate oxidase and Frd have substantial impacts upon ROS levels in *E. coli*. Aspartate oxidase is a minor enzyme but is responsible for 30% of the H_2_O_2_ formed in the aerobic cell (26). Fumarate reductase (Frd) is the terminal enzyme for anaerobic respiration, allowing the cell to use fumarate in place of oxygen as an electron acceptor. It is a close structural homologue of Sdh, but its electronic properties make it much more prone to autoxidation (25). Although expression of the *E. coli* Frd is repressed in oxic environments, the autoxidation of the enzyme becomes a significant event when the bacterium moves from anoxic environments, in which Frd is well expressed, to oxic ones. During the initial period of aeration, intracellular ROS production surges, and the preexisting Frd is responsible (26, 27).

Whereas Frd has an ancillary role in the anaerobic metabolism of *E. coli*, it is a key enzyme in the central pathway of *B. thetaiotaomicron* (Fig. 1B). Thus, we suggested (16) that in this anaerobe the titers of Frd might be especially high and that upon aeration its contribution to ROS formation might be proportionately great. If so, then perhaps even high levels of scavenging enzymes might be inadequate to suppress steady-state levels of ROS, and fumarase inactivation might be the consequence. Subsequently, Meehan and Malamy tested this notion using *Bacteroides fragilis*, a relative that behaves as an obligate anaerobe but has features, such as an oxygen-dependent ribonucleotide reductase, that suggest greater oxygen tolerance. They observed that a *B. fragilis* strain with a mutation in a Frd subunit seemed to release H_2_O_2_ at a diminished rate (28). Their interpretation was that Frd is the primary source of ROS in this bacterium and that this abundant ROS might be involved in poisoning metabolism upon aeration.

In the present study, we confirmed that H_2_O_2_ is produced in aerated *B. thetaiotaomicron* much more rapidly than in *E. coli*. However, biochemical analysis revealed the surprising fact that the Frd of *B. thetaiotaomicron* does not generate either O_2_^−^ or H_2_O_2_. Instead, the iron-sulfur subunit of this enzyme suppresses flavin autoxidation, probably by pulling the electron density away from the solvent-exposed flavin. This example reveals that evolution can modify redox enzymes in a way that suppresses their autoxidation without compromising their physiological function. It is not clear whether this advantage provided the impetus for the electron arrangement of the *B. thetaiotaomicron* enzyme; however, the evolution of enzymes to minimize ROS formation would have been a natural complement to the appearance of other ROS defenses.

## RESULTS

### Hydrogen peroxide is rapidly formed inside *B. thetaiotaomicron* when it is aerated.

**FIG 2:**
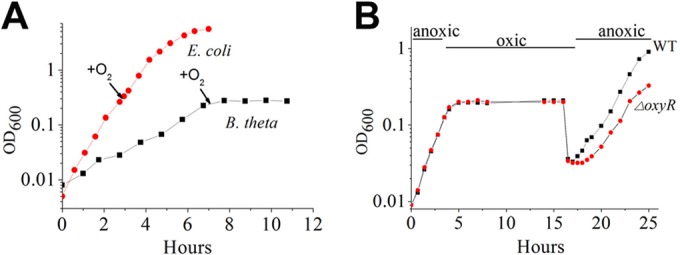
Aeration blocks growth but does not diminish the viability of wild-type *B. thetaiotaomicron.* Cultures were grown in an anaerobic chamber to exponential phase and then transferred to fully oxic environments. The data are representative of three independent experiments.

**FIG 3:**
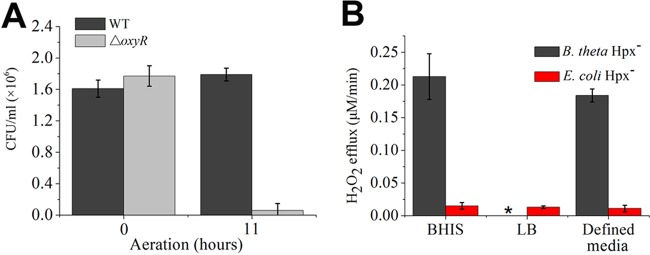
Endogenous H_2_O_2_ formation is much more rapid in aerated *B. thetaiotaomicron* than in aerated *E. coli*. (A) Survival of wild-type and *oxyR* mutant strains after aeration. (B) Mutant strains lacking H_2_O_2_ scavenging enzymes (Hpx^−^) were precultured in the indicated anoxic media, and they were then resuspended in aerobic PBS-glucose or defined media, as described in Materials and Methods. Intracellular H_2_O_2_ production is inferred from the rate of H_2_O_2_ release into the medium. The star denotes that *B. thetaiotaomicron* did not grow in LB medium.

The aeration of *Bacteroides thetaiotaomicron* in BHIS medium caused growth to arrest almost immediately (Fig. 2A). During exposure to oxygen for 11 h, the cells failed to grow but remained fully viable. When anoxia was restored, growth resumed after a short (60- to 90-min) delay (Fig. 2B). This ability to recover quickly from oxygen exposure likely helps *B. thetaiotaomicron* to transit from one host to another. In contrast, after the same period of aeration, *oxyR* mutants exhibited a longer lag of several hours. Closer examination revealed that the *oxyR* mutants suffered a substantial loss of viability during the period of oxygen exposure (Fig. 3A); the lag in subsequent outgrowth reflected the fact that many of the cells were dead.

These results confirm a previous report (17) that *B. thetaiotaomicron*, like its relative *B. fragilis* (29), activates its OxyR response when it is aerated and that this adaptation is critical to its survival. In contrast, the *E. coli* OxyR regulon is not induced upon aeration, and upon aeration, *E. coli oxyR* mutants do not exhibit any survival defect. Since OxyR is activated by hydrogen peroxide, a plausible explanation is that *B. thetaiotaomicron* generates more H_2_O_2_ when oxygen enters its cytoplasm than does *E. coli*. To test this idea, we examined the rate of intracellular H_2_O_2_ formation by measuring the rate at which hydroperoxidase-deficient (Hpx^−^) mutants excrete H_2_O_2_ into the medium. *E. coli* employs two catalases and NADH peroxidase (AhpCF) to degrade hydrogen peroxide, and so *E. coli* Hpx^−^ mutants lack the genes coding for these three enzymes (*katG*, *katE*, and *ahpCF*) (30). *B. thetaiotaomicron* employs a catalase, NADH peroxidase, and two rubrerythrins, and *B. thetaiotaomicron* Hpx^−^ mutants are *katE ahpCF rbr1 rbr2* strains (17). Prior work suggested that Hpx^−^ *B. thetaiotaomicron* excreted H_2_O_2_ at high rates, although the rates were not quantified under the same conditions as for *E. coli*. In the present study, the Hpx^–^ derivatives of both bacteria were cultured in several anoxic complex and defined media, and the bacteria were then shifted to oxic buffer or medium prior to measurements of H_2_O_2_ production. Under identical circumstances, the rate of H_2_O_2_ formation was approximately 10-fold higher for the *B. thetaiotaomicron* strain than for the *E. coli* strain (Fig. 3B). The higher rate of H_2_O_2_ production offers a likely explanation for why OxyR is induced and H_2_O_2_-sensitive enzymes are inactivated when *B. thetaiotaomicron* is aerated, in contrast to *E. coli*.

**FIG 4:**
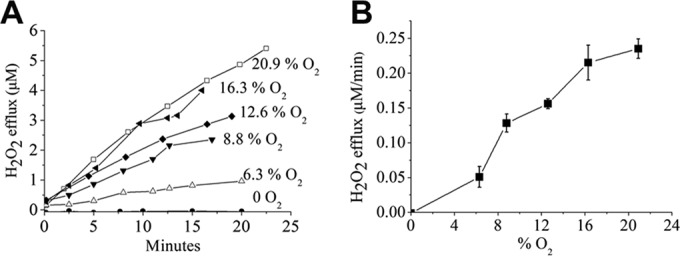
The rate of H_2_O_2_ production in *B. thetaiotaomicron* is proportionate to the ambient O_2_ concentration. Log-phase *B. thetaiotaomicron* Hpx^−^ cells were resuspended in PBS-glucose at an OD_600_ of 0.1. H_2_O_2_ production was monitored at different levels of dissolved O_2_.

The H_2_O_2_ that is formed in aerobic *E. coli* comes from a mixture of sources that have not all been identified. During constant aerobiosis, about one-third of endogenous H_2_O_2_ derives from aspartate oxidase (26), which uses molecular oxygen as an electron acceptor in the first step of nicotinamide biosynthesis. Because this reaction is saturated by moderate levels of oxygen, the rate of H_2_O_2_ formation by this enzyme has a ceiling that is unaffected by further increases in oxygen concentration. In contrast, other endogenous H_2_O_2_ is formed by unknown sources and is generated in proportion to oxygen concentration. This property suggests that this H_2_O_2_ is produced by the adventitious oxidation of redox enzymes by oxygen, therefore displaying chemical rather than Michaelis-Menten kinetics. Similarly, we observed that the high rate of H_2_O_2_ formation by *B. thetaiotaomicron* was proportionate to the concentration of dissolved oxygen (Fig. 4). The mechanistic implication is that H_2_O_2_ is produced by the accidental oxidation of an enzyme(s). In terms of organismal biology, it means that the oxidative stress experienced by the bacterium will be less severe in micro-oxic environments than in fully oxic ones.

The rate of intracellular H_2_O_2_ formation can be derived from the rate at which it accumulates in the growth medium, if one knows the ratio of cell volume to total culture volume (Materials and Methods). In this way, we deduce that if a concentration of cells representing an optical density at 600 nm (OD_600_) of 0.1 causes H_2_O_2_ to accumulate in the medium at a rate of 0.24 μM/min H_2_O_2_ (Fig. 4), then the H_2_O_2_ is formed inside the cells at a rate of approximately 0.1 mM/s. Our previous studies (16) indicated that *B. thetaiotaomicron* consumes glucose at an intracellular rate of slightly more than 1 mM/s. In contrast, based upon protein content and growth rate, we calculate that the fluxes through amino acid biosynthetic pathways are only 10 μM/s; nucleotide synthesis and lipid synthesis are similar, and other pathways are substantially slower. The implication is that central metabolism stands out as having the only pathways with flux capacities compatible with the observed rate of ROS formation. This logic focused our attention upon fumarate reductase.

### *In vivo* evidence does not indicate that fumarate reductase is a major H_2_O_2_ source.

When *E. coli* moves from anoxic to oxic environments, the primary source of H_2_O_2_ is fumarate reductase (Frd) (26). This enzyme is synthesized only during anoxia, but the extant enzyme continues to operate—and releases ROS—when oxygen is infused into the medium. Frd provides a minor pathway of carbohydrate fermentation in *E. coli* (31), but it is central to the anaerobic metabolism of many bacteria, including *B. thetaiotaomicron* (Fig. 1B). We reasoned that *B. thetaiotaomicron* might contain especially high titers of Frd and that Frd might therefore be the source of the high H_2_O_2_ flux after aeration. The most straightforward test of that idea would be to quantify H_2_O_2_ production from a strain that lacked Frd. However, we were unable to recover any *frd* null mutants, despite several attempts and in contrast to our success with all other genes we attempted. We also observed that *B. thetaiotaomicron* grows extremely poorly when hemin is omitted from the medium (data not shown). Hemin is the source of the heme moiety in *B. thetaiotaomicron* Frd, and we suspect that the requirement for heme in growth medium reflects the importance of this enzyme to anaerobic metabolism. For these reasons, it was not possible to genetically test the hypothesis that Frd produces most of the intracellular H_2_O_2_.

As an alternative, we appraised the effect of fumarate supplementation upon H_2_O_2_ release. In principle, the *B. thetaiotaomicron* Frd is especially vulnerable to autoxidation because the fumarase of this bacterium loses activity upon aeration. The inactivity of fumarase blocks the formation of intracellular fumarate, leaving Frd without a natural electron acceptor (Fig. 1B). When other redox enzymes are deprived of their acceptors, they are much more prone to pass electrons to oxygen (27). Therefore, we provided fumarate as a supplement in the medium. Bacteria that employ fumarate as a terminal electron acceptor are usually able to import it, and indeed the *B. thetaiotaomicron* genome encodes two membrane proteins homologous to the *E. coli* DcuA and DcuB fumarate importers (32). Previous work confirmed that exogenous fumarate is imported and reduced by Frd (16). However, fumarate supplements did not diminish the rate of intracellular H_2_O_2_ production (see Fig. S1A in the supplemental material). This result did not support the notion that Frd is a major source of H_2_O_2_ in *B. thetaiotaomicron*.

10.1128/mBio.01873-16.1 Figure S1  *B. thetaiotaomicron* fumarate reductase is not a source of ROS *in vivo* or *in vitro*. (A) Cellular H_2_O_2_ production was not diminished when 0.5 mM fumarate was included in the medium. This concentration exceeds by 10-fold the *K*_*m*_ values of members of the Dcu fumarate transport family and can provide sufficient fumarate to enable fumarate-dependent respiration. (B and C) Rates of O_2_^−^ (B) and H_2_O_2_ (C) formation by inverted membrane vesicles incubated with various concentrations of succinate. Squares, vesicles prepared from *E. coli*; circles, vesicles from *B. thetaiotaomicron*; triangles, vesicles from *B. fragilis*. Download Figure S1, TIF file, 2.7 MB. Copyright © 2017 Lu and Imlay. 2017 Lu and Imlay This content is distributed under the terms of the Creative Commons Attribution 4.0 International license.

### The *B. thetaiotaomicron* Frd, unlike the *E. coli* enzyme, does not produce ROS *in vitro*.

The previous result surprised us. We then examined the behavior of the Frd enzyme in inverted membrane vesicles. The vesicles were prepared by French press. The physiological reaction of Frd is to transfer electrons from quinones to fumarate (Fig. 1A), thereby generating succinate, but the reaction is reversible both *in vitro* and *in vivo*. We found that the succinate:quinone oxidoreductase activities were quantitatively similar in anoxically grown *E. coli* and *B. thetaiotaomicron*, indicating similar titers of Frd, contrary to our expectation (see Fig. S2 in the supplemental material). Assays of NADH:fumarate reductase activity confirmed that result (not shown).

10.1128/mBio.01873-16.2 Figure S2  The fumarate reductase activities of *E. coli* and *B. thetaiotaomicron* membranes are similar. Membranes were prepared from cells grown in defined anaerobic glucose media, as described in Materials and Methods. Succinate:plumbagin oxidoreductase activities were assayed as a measure of Frd content. Download Figure S2, TIF file, 1.4 MB. Copyright © 2017 Lu and Imlay. 2017 Lu and Imlay This content is distributed under the terms of the Creative Commons Attribution 4.0 International license.

**FIG 5:**
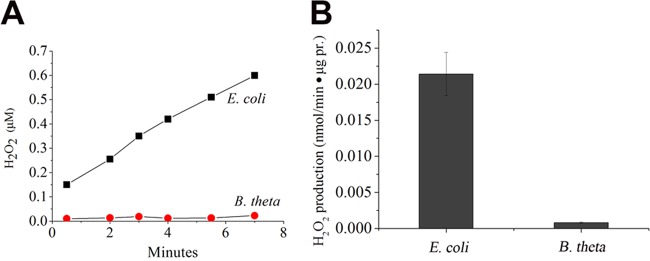
In membrane vesicles, the *E. coli* Frd produces H_2_O_2_, but the *B. thetaiotaomicron* Frd does not. (A) Representative time course during incubation with 0.4 mM succinate. (B) Rates of H_2_O_2_ production at 0.4 mM succinate. pr., protein. The fumarate reductase activities (succinate:plumbagin oxidoreductase activities) of the membranes were approximately equivalent (Fig. S2).

**FIG 6:**
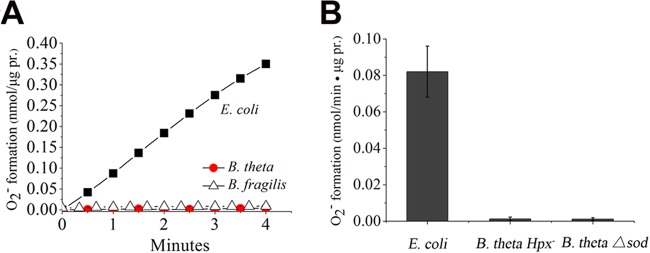
The *E. coli* Frd produces O_2_^−^, but the *B. thetaiotaomicron* Frd does not. (A) Representative time course during incubation of vesicles with 0.4 mM succinate. pr., protein. (B) Rates of O_2_^−^ production at 0.4 mM succinate. Membranes from a *B. thetaiotaomicron* SOD^−^ mutant also produced no O_2_^−^, confirming that the lack of O_2_^−^ detection was not due to contaminating SOD.

When *E. coli* vesicles were incubated with succinate in oxic buffer, both profuse H_2_O_2_ and superoxide were formed (Fig. 5 and Fig. 6; Fig S1B and C). As was reported before, the formation of this ROS depended upon the presence of Frd (see Table S1). In surprising contrast, despite exhibiting ample succinate:quinone reductase activity, the succinate-treated *B. thetaiotaomicron* vesicles did not generate any significant amount of either oxidant (Fig. 5 and 6). The *E. coli* enzyme characteristically produced less superoxide at higher doses of succinate, since the more-reduced enzyme releases electrons to oxygen in pairs rather than singly; at very high doses, succinate prevents all ROS formation by occluding the flavin. The *B. thetaiotaomicron* enzyme did not release either species at any concentration of succinate (Fig. S1B and C).

10.1128/mBio.01873-16.7 Table S1  Fumarate reductase is the source of succinate-dependent ROS formation by *E. coli* membrane vesicles. Download Table S1, DOC file, 0.1 MB. Copyright © 2017 Lu and Imlay. 2017 Lu and Imlay This content is distributed under the terms of the Creative Commons Attribution 4.0 International license.

**FIG 7:**
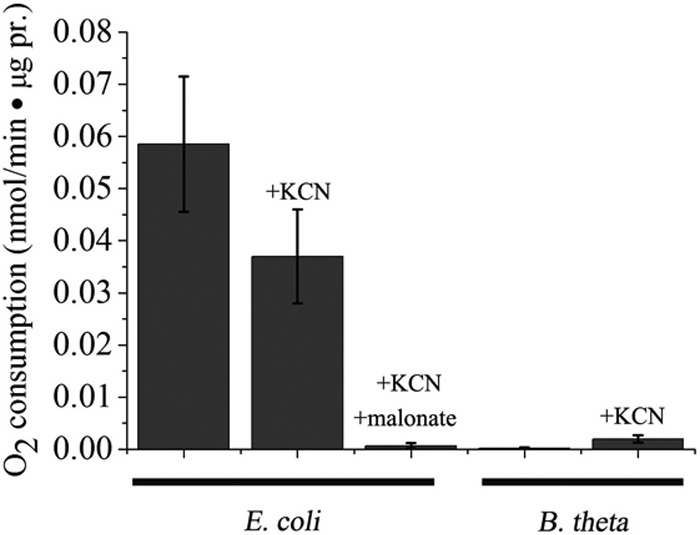
Cyanide-resistant O_2_ consumption by inverted vesicles confirms that *E. coli* Frd produces ROS but *B. thetaiotaomicron* Frd does not. Reaction mixtures contained 0.4 mM succinate as the electron donor and 3 mM KCN to block oxygen consumption by cytochrome oxidase. Where indicated, 3 mM malonate was added to block the access of oxygen to the flavin of Frd.

The contrasting autoxidation behaviors of the two enzymes were also apparent from measurements of oxygen consumption. When *E. coli* membranes were incubated with succinate, oxygen was consumed even if cytochrome oxidase was blocked by cyanide (Fig. 7). The rate of oxygen consumption matched the rate of H_2_O_2_ release. Yet the *B. thetaiotaomicron* membranes did not consume oxygen at all (<8% the rate of the *E. coli* membranes).

In a prior publication (26), we proposed that obligate anaerobes might minimize ROS formation by Frd through the actions of cytochrome *bd* oxidase. When oxygen is present, this oxidase competes with Frd for reduced quinones and thereby diminishes ROS production by the *E. coli* enzyme. However, this arrangement does not explain the lack of O_2_^−^ and H_2_O_2_ deriving from the *B. thetaiotaomicron* Frd, as virtually no oxygen consumption occurred even when cyanide was not provided to inhibit the oxidase (Fig. 7). In separate experiments, we confirmed that *B. thetaiotaomicron* consumes oxygen upon aeration, but the rate is undiminished in cytochrome *bd* oxidase mutants. Instead, rubredoxin:oxygen oxidoreductase (Roo), a soluble enzyme, is responsible (see Table S2 in the supplemental material). We infer that under our anoxic culturing conditions, the cytochrome *bd* oxidase was not expressed and therefore had no effect on Frd behavior.

10.1128/mBio.01873-16.8 Table S2  O_2_ consumption by aerated *B. thetaiotaomicron*. Download Table S2, DOC file, 0.1 MB. Copyright © 2017 Lu and Imlay. 2017 Lu and Imlay This content is distributed under the terms of the Creative Commons Attribution 4.0 International license.

During normal metabolism, electrons flow from NADH through the menaquinone pool to Frd (Fig. 1A). This electron transfer pathway is active in inverted vesicles (see Fig. S3A in the supplemental material). The addition of cyanide to *E. coli* vesicles diminished the rate of NADH oxidation by inhibiting the cytochrome oxidases, but it did not limit NADH oxidation entirely, because adventitious electron transfer from reduced chain components to oxygen persisted. Much of this residual flux was due to Frd autoxidation, as it was blocked by malonate, a potent competitive inhibitor that binds opposite the flavin and impedes the approach of oxygen. With *B. thetaiotaomicron* vesicles, only a modest amount of oxygen consumption was detected, and it was resistant to both cyanide and malonate. This NADH consumption was likely due to the autoxidation of NADH dehydrogenase (4). The data indicate that Frd does not autoxidize even when electrons arrive through the quinone pool. That conclusion was further supported by assays of H_2_O_2_ production (Fig. S3B). Importantly, these data indicate not only that Frd is not the source of the outsized H_2_O_2_ formation in *B. thetaiotaomicron*, but also that other components of the electron transport chain are not the source, either.

10.1128/mBio.01873-16.3 Figure S3  Cyanide-resistant NADH oxidation by membrane vesicles reflects electron leakage to oxygen by *E. coli* Frd but not *B. thetaiotaomicron* Frd. (A) NADH oxidation. (B) H_2_O_2_ formation. Reaction mixtures contained inverted vesicles from Hpx^−^ strains, 120 μM NADH as the electron donor, 3 mM KCN to inhibit cytochrome oxidase, and (where indicated) 2.5 mM malonate to block access of oxygen to the flavin of Frd. Residual NADH oxidation (A) and H_2_O_2_ production (B) are caused by adventitious electron transfer from chain components to O_2_. Malonate inhibits oxidation at the Frd flavin site. Download Figure S3, TIF file, 1.7 MB. Copyright © 2017 Lu and Imlay. 2017 Lu and Imlay This content is distributed under the terms of the Creative Commons Attribution 4.0 International license.

The previous study of *B. fragilis* showed that a mutation that knocked out *frdC* diminished the rate of H_2_O_2_ release from whole cells by about 40%; an inference was drawn that Frd might be the source of the lost H_2_O_2_. However, *B. fragilis frd* mutants grow at only 30% the rate of their wild-type parent (33), which raised the alternative possibility that slow metabolism was the cause of the drop. To look more directly, we prepared membranes from the same *B. fragilis* strain and tested the ability of its Frd to generate superoxide. Like the *B. thetaiotaomicron* enzyme, with which it shares *ca*. 90% identity, the *B. fragilis* Frd did not generate any detectable superoxide (Fig. 6; Fig.S1B).

### The identity of the iron-sulfur subunit determines the rates of Sdh/Frd autoxidation.

A key structural distinction between the Frd enzymes of *E. coli* and *B. thetaiotaomicron* is that the latter enzyme is among the members of the complex II family (34) that have two heme binding sites in the membrane anchor subunit FrdC (Fig. 1; see Fig. S4 in the supplemental material). In that respect it resembles *E. coli* Sdh, which also includes a heme moiety (35). In contrast, the *E. coli* Frd instead employs two smaller transmembrane subunits that lack heme-binding ligands and do not incorporate heme as a cofactor (36). Redox calculations led Yankovskaya et al. to propose that the heme cofactor of Sdh would diminish electron occupancy of its flavin cofactor, which might diminish ROS formation (35). Indeed, Sdh generates far less H_2_O_2_ and O_2_^−^ than does Frd (25).

10.1128/mBio.01873-16.4 Figure S4  Alignments of *frdC* or *sdhC* subunits revealing heme ligands. Axial histidine residues that coordinate hemes are shown in gray for those that bind distal heme b and in red for those that bind proximal heme b, as identified by the crystal structure of the *Wolinella succinogenes* Frd (66). *E. coli* Sdh has a single (proximal) heme with axial histidines provided by SdhC (shown above) and SdhD (not shown). Sequence analysis identified the BT_3053–3055 operon as the sole *frd* operon in *B. thetaiotaomicron* (Btheta). It contains cytochrome *b* (BT_3053), flavin (BT_3054), and iron-sulfur (BT_3055) subunits. Accession numbers for the other aligned genes are as follows: *Bacteroides fragilis frdC*, BF9343_4224; *Desulfovibrio gigas frdC*, WP_021759412; *E. coli sdhC*, EG10933; and *W. succinogenes frdC*, WP_011138740. Download Figure S4, TIF file, 2.6 MB. Copyright © 2017 Lu and Imlay. 2017 Lu and Imlay This content is distributed under the terms of the Creative Commons Attribution 4.0 International license.

**FIG 8:**
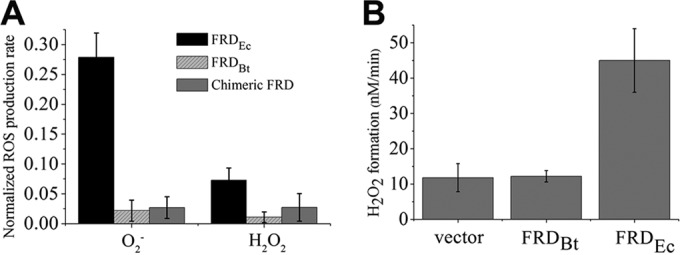
The absence of ROS production by *B. thetaiotaomicron* FRD does not derive from the lipid composition or the nature of its flavoprotein subunit. (A) Cell membranes were prepared from *E. coli* Hpx^−^ cells, from cells of the the *E. coli* Hpx^−^ Δ*frd* mutant carrying a plasmid expressing *B. thetaiotaomicron* wild-type Frd (*frdCBA*), and from cells of the *E. coli* Hpx^−^ Δ*frd* mutant expressing the chimeric Frd (*E. coli frdA* plus* B. thetaiotaomicron frdBC*). The rates of *in vitro* O_2_^−^ and H_2_O_2_ production are normalized to the succinate:plumbagin oxidoreductase activities. Reaction mixtures contained 0.4 mM succinate; 3 mM KCN was included in measurements of O_2_^−^ formation. (B) Only *E. coli* Frd generates H_2_O_2_ *in vivo*. The *E. coli* Hpx^−^ Δ*frdABCD* strain LC126 was transformed with empty vector, plasmid pfrd(CAB)_Bt_ expressing *B. thetaiotaomicron* Frd, or plasmid pH3 expressing *E. coli* Frd. Fumarate reductase was induced in anoxic lactose-Casamino Acids medium, and H_2_O_2_ excretion was then quantified after dilution into oxic glucose buffer (see Materials and Methods).

To test the significance of this difference, the *frdCAB* operon of *B. thetaiotaomicron* was expressed in a Δ*frd* Δ*sdh* mutant strain of *E. coli*. The vesicles exhibited robust succinate:quinone oxidoreductase activity, but again the *B. thetaiotaomicron* Frd did not generate either O_2_^−^ or H_2_O_2_ (Fig. 8A). Moreover, the expression of *E. coli* Frd in Hpx^−^ cells caused substantial excretion of H_2_O_2_ when the cells were aerated, but expression of the *B. thetaiotaomicron* enzyme did not (Fig. 8B). These results confirmed that its nonautoxidizability is a trait of the *B. thetaiotaomicron* Frd itself rather than of the *B. thetaiotaomicron* lipid or quinone environment. To dissect the enzyme further, we attempted to create chimeric enzymes that contained mixtures of the subunits from the two bacterial Frd enzymes. Only the combination of the *E. coli* FrdA subunit and the *B. thetaiotaomicron* FrdB and -C subunits provided active enzyme (see Fig. S5 in the supplemental material). This hybrid enzyme also did not autoxidize (Fig. 8A). Thus, the suppression of Frd autoxidation behavior is not due to differences in the FrdA subunit, which is the actual site of electron transfer to oxygen, but rather to the effects of the FrdB/C subunits upon it. These data fit the model (25, 35) that electron withdrawal by the iron-sulfur cluster and/or heme moieties can minimize autoxidation from the flavin adenine dinucleotide (FAD) site.

10.1128/mBio.01873-16.5 Figure S5  Expression and oxidation of *B. thetaiotaomicron/E. coli* Frd hybrid and mutant proteins in *E. coli*. (A) Diagram of the constructed plasmids expressing *B. thetaiotaomicron* Frd and chimeric Frd proteins. (B) Succinate:plumbagin (PB) reductase activities of Frd in cell membranes, normalized to total membrane protein. Frd hybrid and mutant proteins were prepared from anaerobic KM7 (△*sdh* △*frd*) cells. Only the heterologous *B. thetaiotaomicron* FrdABC protein and the FrdA_Ec_-FrdBC_Bt_ hybrid construct provided active forms of Frd. No activity was recovered from constructs with mutations in the *frdC* histidine residues that are predicted to provide axial ligands to the proximal heme. (C) Covalent flavin fluorescence detection. The FAD covalently bound to Frd was visualized by exposing the unstained SDS gel to UV transillumination (left panel). UV fluorescence was quantified by the Quantity One system (Bio-Rad) (right panel). Lanes contain 275-μg cell membranes from anaerobic cultures. Lane 1, KM7-pfrd(CAB)_Bt_; lane 2, KM7-pfrd(CB)_Bt_(A)_Ec_; lane 3, *E. coli* Hpx^−^ (LC106); lane 4, KM7 (Δ*frd*). M, marker, consisting of Bio-Rad protein dual-color standards (the marked band showed strong UV fluorescence). Nd, not detected. (D) Succinate:plumbagin reductase activities of the samples, normalized to covalent FAD content. (E) The removal of the heme from Sdh does not trigger Frd-level O_2_^−^ formation. Cell membranes were prepared from quinone-deficient strains expressing wild-type *E. coli* Frd (KM8 with pH3 plasmid), wild-type *E. coli* Sdh (KM8 with pFAS plasmid), or heme-free *E. coli* Sdh (KM8 with pFAS plasmid encoding *E. coli* SDH-H84Y). Data represent O_2_^−^ production normalized to succinate:ferricyanide reduction rates. Note the break in the *y*-axis scale. Download Figure S5, TIF file, 2.8 MB. Copyright © 2017 Lu and Imlay. 2017 Lu and Imlay This content is distributed under the terms of the Creative Commons Attribution 4.0 International license.

To specifically test the impact of the heme moieties upon Frd autoxidation, it would be ideal to eliminate the heme binding sites of the *B. thetaiotaomicron* C subunit. However, we were unable to recover active enzyme from *E. coli* strains expressing such a mutant construct (Fig. S5). It is likely that the hemeless C subunit was unstable. As an alternative, we tested the impact of the heme upon the autoxidation rate of *E. coli* Sdh. Tran et al. generated such a mutant, and in their own studies, they did not observe any increase in Sdh oxidation during succinate respiration (37, 38). To examine this issue more closely, we expressed both the wild-type and heme-deficient enzymes in a quinoneless strain of *E. coli*, thereby ensuring that the rate of electron flow to the quinone pool did not have any impact upon autoxidation. Quite clearly, the removal of heme did not convert Sdh to high ROS production in the fashion of Frd (Fig. S5E). We think this result can likely be extrapolated to *B. thetaiotaomicron* Frd. Although that enzyme additionally contains a distal heme that Sdh lacks, the distal hemes in Frd di-heme enzymes typically lie at low potentials (34) and should have minimal impact as electron sinks. Collectively, these data indicate that the pace of flavin autoxidation in this enzyme family is determined by the nature of the iron-sulfur subunit rather than by either the structure of the flavoprotein subunit or the presence of heme cofactors.

### What is the primary source of ROS in *B. thetaiotaomicron*?

Thus, *B. thetaiotaomicron* generates high levels of intracellular oxidants when it encounters oxygen, but the source is neither Frd nor any of the other components of the membrane-bound respiratory chain. One alternative was the series of redox enzymes that deliver electrons to Roo, the soluble oxygen reductase. Those electrons flow from NADH through rubredoxin to Roo (17, 39, 40). When rubredoxin was deleted from the non-scavenging strain, those mutants (*rd*) generated 25% less H_2_O_2_ than did the rubredoxin-proficient strain (see Table S3 in the supplemental material). NADH:rubredoxin oxidoreductase (NROR) is one source of those electrons, but not the sole one. Both NROR- and Roo-deficient mutants exhibited wild-type rates of H_2_O_2_ formation. Thus, the predominant sources of ROS remain unknown.

10.1128/mBio.01873-16.9 Table S3  H_2_O_2_ generation is diminished in rubredoxin-deficient strains. Download Table S3, DOC file, 0.1 MB. Copyright © 2017 Lu and Imlay. 2017 Lu and Imlay This content is distributed under the terms of the Creative Commons Attribution 4.0 International license.

## DISCUSSION

### Rapid ROS formation precludes aerobic growth but is not lethal.

Dosimetric studies showed that *E. coli* expresses just enough scavenging activity to avoid being poisoned by its endogenous O_2_^−^ and H_2_O_2_ (12). When the steady-state levels of these oxidants were elevated more than 3-fold, its oxidant-sensitive enzymes lost activity, and growth slowed. Against this backdrop, it is striking that aeration drives *Bacteroides thetaiotaomicron* to generate 10-fold more ROS than does *E. coli*. Further, *B. thetaiotaomicron* does not compensate with a proportionate increase in the titers of its scavenging enzymes; in fact, we determined that its SOD levels (2 U/mg) are much lower than those in either aerobic (16 U/mg) or even anaerobic (5 U/mg) *E. coli* cells (see Fig. S6 in the supplemental material). In conjunction with the higher rate of ROS formation, the implication is that the steady-state level of superoxide in fully aerated *B. thetaiotaomicron* may exceed that of *E. coli* by >25-fold. This situation provides a good explanation for why *B. thetaiotaomicron* aeration is followed by the progressive loss of fumarase activity, the collapse of the succinate/propionate branch of its fermentation, and the cessation of growth (16).

10.1128/mBio.01873-16.6 Figure S6  SOD activities of wild-type *B. thetaiotaomicron* or *E. coli*. *E. coli* samples were prepared from aerobic or anaerobic cultures. Download Figure S6, TIF file, 1.9 MB. Copyright © 2017 Lu and Imlay. 2017 Lu and Imlay This content is distributed under the terms of the Creative Commons Attribution 4.0 International license.

This vulnerability is one factor that constrains *B. thetaiotaomicron* to life in anoxic environments. Nevertheless, the bacterium tolerates aeration well enough to move between hosts. Furthermore, if the human gut is perforated by physical trauma, *Bacteroides* species are able to spread into the erstwhile oxic peritoneum and form life-threatening abscesses (41). These events manifest the theme that all anaerobes must withstand occasional oxygen exposure. How they manage to do so is a focus of substantial research activity.

Several features sustain *B. thetaiotaomicron* when it enters oxic environments. The fact that ROS formation is proportionate to oxygen level (Fig. 4) has the consequence that stress is lessened in tissues, where oxygen levels are at least 5-fold lower than in air-saturated fluids. Second, while the ROS can inactivate enzymes and impair regular metabolism, they do not generate life-threatening DNA lesions (Fig. 3A), because the OxyR-mediated induction of iron storage proteins defuses the possibility of Fenton chemistry (42). Finally, by using its respiration-linked cytochrome *bd* oxidase and cytoplasmic rubredoxin:oxygen oxidoreductase, *B. thetaiotaomicron* presumably can gradually clear oxygen from the local microenvironment, allowing the bacterium to repair its damaged enzymes and to restore its full metabolic capacity. Nevertheless, ROS production places a limit upon the oxygen level that allows the resumption of metabolism. We were surprised to discover that the fumarate reductase of *B. thetaiotaomicron*, which we and others anticipated would be the major source of ROS, has instead acquired a structure that avoids any detectable ROS formation at all.

### Why does not *B. thetaiotaomicron* fumarate reductase generate ROS?

The complex II family of enzymes—aspartate oxidase, fumarate reductase, and succinate dehydrogenase—has come under especially close study because the autoxidation of these enzymes has important physiological consequences. Previous studies suggested a model for how their redox structures create differences in their autoxidation behaviors. Aspartate oxidase is the simplest member. It is comprised of a single flavoprotein subunit (43), whose flavin is alternately reduced by aspartate and oxidized (in anoxic cells) by fumarate. Hence it is a fumarate reductase. However, in aerobic cells, the fumarate level falls too low, and oxygen itself receives the electrons with stoichiometric conversion to H_2_O_2_ (26). Kinetic analysis showed that the oxidation step is not saturated by O_2_, which reveals that the enzyme has no authentic O_2_ binding site. Thus, aspartate oxidase really is an aspartate:fumarate oxidoreductase whose turnover is sustained in aerobic cells by the chemical oxidizability of its exposed flavin. This switch in electron acceptor is unique and remarkable.

The flavoprotein subunit of respiratory fumarate reductase is tethered to the cell membrane by the FrdB iron-sulfur wire and the FrdC/D integral membrane subunits (Fig. 1A). The latter also comprise a quinone binding site. In anaerobic cells, electrons arrive from the quinone pool of the respiratory chain, flow through the iron-sulfur centers to the flavin, and again are transferred to fumarate as the physiological acceptor. However, when *E. coli* cells transit to an oxic environment, oxygen can again accept electrons from the reduced flavin. Oxygen is a diradical, so the electron transfer necessarily occurs through consecutive single-electron events (44). When Frd is oxidized, the predominant product is O_2_^−^ rather than H_2_O_2_ (25), because after transfer of the first electron from FADH_2_, the second electron can be sequestered on a nearby iron-sulfur cluster. The initial O_2_^−^ diffuses out of the active site, and the second electron is ultimately transferred to oxygen in a second oxidation event. The O_2_^−^ that Frd forms is more hazardous than the H_2_O_2_ that aspartate oxidase forms, because O_2_^−^ damages vulnerable enzymes more quickly.

The failure of *B. thetaiotaomicron* Frd to autoxidize like *E. coli* Frd is reminiscent of the behavior of *E. coli* Sdh. Sdh shares the same flavin-iron-sulfur linkage as Frd, but its clusters I and III sit at a higher potentials (+10 mV and +65 mV [45, 46]) than those of Frd (−35 mV and −65 mV [47, 48]) and in the divalently reduced enzyme are expected to pull the electron density away from the flavin. A much slower pace of flavin oxidation is the expected result. Yandovskaya et al. suggested that this effect might be further enhanced by the Sdh heme (35), but data from Tran et al. (37, 38) and from Fig. 8 show that this is not necessary.

Against this backdrop, a reasonable explanation for the nonoxidizability of *B. thetaiotaomicron* Frd is that it, like Sdh, may have higher-potential clusters that suppress flavin autoxidation. Measurements will be necessary to confirm the idea; other explanations are possible. However, the fact that *B. thetaiotaomicron* FrdB/C subunits suppressed the autoxidation of *E. coli* FrdA certainly fits the model.

What is new about this observation is that, unlike *E. coli* Sdh, the *B. thetaiotaomicron* Frd retains its role as a fumarate reductase. That is, the suspected shift of electron density away from its flavin does not hinder physiological electron flow to the fumarate site. Although at first blush this outcome might seem surprising, further consideration of redox dynamics would argue that electron distribution on an enzyme does not need to correlate rigidly with the direction of its flow. Electron exchange between the redox moieties in all of these enzymes is certainly extremely fast relative to the lifetime of either enzyme-substrate complex; thus it is unlikely that a shift in electron density will create a bottleneck in the catalytic cycle, which involves the much slower steps of substrate binding and product release (49). The same idea can be invoked to explain why catalysis in either direction would be unimpeded by the requirement that electrons flow across the very-low-potential cluster II (−250 to −300 mV) (50). Yet the redistribution of electron density from one side of the enzyme to the other will strongly affect the pace of electron transfer to oxygen, since the collision of oxygen with the flavin is a momentary elastic event.

There is an interesting parallel between *B. thetaiotaomicron* Frd and class I ribonucleotide reductases, which evolved to reduce ribonucleotides in the aerobic world. Ribonucleotide reductases employ a cysteinyl radical to abstract an electron from ribonucleotides. Such radicals are extremely vulnerable to oxidation by molecular oxygen—but in the class I enzymes, this calamity is avoided because the radical is predominantly localized on an electronically linked tyrosine residue, which is buried in the protein and thereby shielded from oxygen. When substrate binds, resonance relocates the radical to the active-site cysteine at a rate sufficient for robust turnover. Similarly, in *B. thetaiotaomicron* Frd—and *E. coli* Sdh—the localization of the electron pair on iron-sulfur clusters away from the exposed flavin may be an adaptation that circumvents inappropriate autoxidation.

Interestingly, some complex I (NADH dehydrogenase) isozymes exhibit an extra redox moiety that, like the heme of Sdh, had been proposed to suppress ROS formation by electron withdrawal. NADH dehydrogenase uses a long iron-sulfur wire to move electrons from the solvent-exposed flavin to quinone-reduction site, but an additional cluster (N1a) lies adjacent to the flavin but outside of the wire itself. It was hypothesized that during turnover, the extra cluster might momentarily sequester an electron from the flavin, minimizing its flavosemiquinone content and thereby diminishing ROS production (51, 52). However, conversion of the N1a cluster to a nonreducible low-potential center did not alter the autoxidation rate of the isolated enzyme (53). Thus, for the moment the disparity in autoxidation behaviors of *B. thetaiotaomicron* and *E. coli* Frd enzymes is a singular demonstration that some redox enzymes can acquire structures that suppress their tendency to contribute to oxidative stress. This is a logical complement to the evolutionary appearance in some organisms of oxidant-resistant enzymes, such as special isozymes of fumarase (54), dihydroxyacid dehydratase (55), hydrogenase (56), and pyruvate:ferredoxin oxidoreductase (57).

Finally, we note that the identity of the residual ROS source in *B. thetaiotaomicron* remains unknown. The *in vitro* data indicated that none of the components of the anaerobic respiratory circuit is likely to be responsible, as ROS leakage from the chain *in vitro* was not markedly different from that from the *E. coli* chain. Prior analyses have shown that, aside from Frd, the *E. coli* chain is not a major ROS contributor to that bacterium (58). In *B. thetaiotaomicron*, the soluble rubredoxin-dependent electron chain seems to be a minor source. An attractive candidate for the residual ROS might be the ferredoxin-mediated flow of electrons from pyruvate to hydrogenase, since this high-volume pathway involves a series of metal centers that operate at low potential near enzyme surfaces. It will be important to identify the main *B. thetaiotaomicron* ROS source, since it is likely responsible for the deactivation of fumarase upon aeration and in part for the consignment of the bacterium to hypoxic and anoxic habitats.

## MATERIALS AND METHODS

### Chemicals.

Most chemicals were purchased from Sigma. Amplex UltraRed was bought from Invitrogen. Horseradish peroxidase (HP), horse heart cytochrome *c*, and *E. coli* iron-containing superoxide dismutase were all Sigma products.

### Cell growth and media.

Anaerobic cultures were grown at 37°C in an anaerobic glove box (Coy Laboratory Products) containing 85% N_2_, 10% H_2_, and 5% CO_2_. Defined medium for *E. coli* contained minimal A salts (59), 0.2% glucose, 0.2% Casamino Acids, 0.5 mM tryptophan, and 5 μg/ml thiamine. BHIS medium and defined glucose medium for *B. thetaiotaomicron* were made as described previously (60). *B. fragilis* was also grown in BHIS medium. Media for anaerobic cultures were autoclaved and then moved into the anaerobic chamber and degassed for at least 24 h before use. Plasmids were transformed by the CaCl_2_ method into *E. coli* Hpx^−^ (LC106 or LC126) or *ubiA menA* (KM8) strains inside the anaerobic chamber. To ensure that quinone mutants had not reverted during cell growth for the preparation of membranes, some harvested cells were streaked onto aerobic LB medium to confirm that the colonies remained tiny after 2 days of growth.

Cell growth was monitored by measurement of OD_600_. Prior to aeration of previously anaerobic cultures, log-phase cells were grown anoxically from an OD_600_ of 0.005 to an OD_600_ of approximately 0.2. Cells were centrifuged, and the pellets were then resuspended in warm aerobic medium. The viability of aerated *B. thetaiotaomicron* was tracked by transferring cells back to the anaerobic chamber and plating them on anoxic BHIS plates. Colonies were counted after 3 days.

The strains and plasmids used in this study are listed in Table S4 in the supplemental material. The *B. thetaiotaomicron* strains were derived from BT5482 △*tdk*, as described previously (61). The deletion of genes was performed by standard methods (61), and deletions were confirmed by sequencing or genome PCR. A low-copy-number plasmid (pWKS30) was used to construct plasmids that express chimeric fumarate reductase enzymes in *E. coli*. Genes encoding the three subunits were cloned behind the *lac* promoter. The ribosome binding site (RBS) of *E. coli gapA* was inserted upstream of each gene. Construction was confirmed by digestion and/or DNA sequencing.

10.1128/mBio.01873-16.10 Table S4  Strains and plasmids. Download Table S4, DOC file, 0.1 MB. Copyright © 2017 Lu and Imlay. 2017 Lu and Imlay This content is distributed under the terms of the Creative Commons Attribution 4.0 International license.

Plasmid pfrd(CAB)_Bt_ was used as the template for site-directed mutagenesis of *B. thetaiotaomicron frdC* (H178L, H178Q, or H178Y). After mutagenesis PCR, DpnI was used to digest the template plasmid for 1 h at 37°C. Oligonucleotides were synthesized by Eurofins MWG Operon (United States). Mutant alleles were confirmed by sequencing (ACGT, Inc., USA).

The plasmids were transformed into the *E. coli* △*sdh* △*frd* strain (KM7) or the *E. coli* Hpx^−^ △*frd* strain (LC126). Cells were grown in minimal medium containing lactose as the sole carbon source and inducer before membrane vesicles were prepared, as described below.

### Preparation of inverted membrane vesicles.

Membrane vesicles were prepared by standard methods (27). Cells were grown under anaerobic conditions from an *A*_600_ of 0.005 to 0.2 to 0.3 and then were washed in cold potassium phosphate buffer (50 mM [pH 7.8]), suspended in the same buffer at 1% of the original volume, and lysed by French press. Cell debris was removed by centrifugation (4°C, 20,000 × *g* for 15 min), and the supernatant was then diluted 5-fold in buffer and centrifuged in an ultracentrifuge (4°C, 100,000 × *g* for 1.5 h). The supernatant was discarded, and the resulting pellet was resuspended in the same potassium phosphate buffer. The ultracentrifugation step was then repeated, and the vesicles were finally suspended in ~1% of the original culture volume in potassium phosphate buffer. Vesicles were stored on ice. Protein concentrations were measured using the Coomassie blue reagent from Thermo-Fisher.

### Measurements of oxygen consumption.

Oxygen consumption by respiring vesicles was measured using a Clark oxygen electrode. Vesicles were added to KP_i_ buffer that had been prewarmed in a 37°C water bath. Succinate (0.4 mM) and KCN (3 mM) were added to detect oxygen consumption that was independent of electron flow through cytochrome oxidase; this rate represented production of O_2_^−^ and H_2_O_2_ by adventitious electron transfer to oxygen upstream in the respiratory chain. Where indicated, 3 mM malonate was added as a specific inhibitor of fumarate reductase. Measurements were performed at 37°C.

Subunit A of fumarate reductase was detected after electrophoresis of membrane samples containing 275 μg protein on SDS-polyacrylamide gels (62). The gels were exposed to UV transillumination, and the UV fluorescence of the bound flavin was quantified by a Quantity One system (Bio-Rad).

### Measurements of H_2_O_2_ formation.

The formation of H_2_O_2_ by aerated cells was measured. Hpx^−^ cells that had been cultured into log phase in anaerobic rich medium were harvested by centrifugation, washed with phosphate-buffered saline (PBS) buffer (pH 7.2), and resuspended to an OD_600_ of 0.1 in warm PBS buffer containing 0.05% glucose. The OD_600_ was around 0.1. Cells that had been grown in anaerobic basic media were resuspended in minimal A medium with 0.05% glucose and 0.01% Casamino Acids or in defined medium with glucose but without hemin and cysteine. Hemin and cysteine can interfere with H_2_O_2_ formation. The resuspended cells were shaken at 37°C under room air. At intervals, samples were removed, and their H_2_O_2_ content was determined by Amplex Red-horseradish peroxidase (HRP) analysis.

The dependence of H_2_O_2_ formation upon solution oxygen content was determined in a similar way. *B. thetaiotaomicron* Hpx^−^ cells were cultured from an OD_600_ of 0.01 to 0.25 in anoxic BHIS medium. Cells were washed and resuspended to an OD_600_ of 0.1 in anoxic PBS (pH 7.2) buffer containing 0.2% glucose; the medium had been presaturated by a gas mixture of N_2_-air and maintained in a 37°C water bath. At intervals, samples were removed, and H_2_O_2_ was quantified by the Amplex Red-HRP method. The gas mixture was established by mixing gas flow from nitrogen and oxygen cylinders at a Y intersection; the gas stream then was bubbled through a water trap (to ensure hydration) and finally through the cell culture.

The rate of H_2_O_2_ excretion by cells can be used to derive the rate of intracellular H_2_O_2_ production. We previously determined that 1 ml of *E. coli* cells at an OD_600_ of 1 comprises 0.5 μM cytoplasm (63); therefore, for the cultures at an OD_600_ of 0.1 used in H_2_O_2_ measurements, the ratio of culture volume to cytoplasmic volume is 20,000:1. Hence an excretion rate of 0.24 μM H_2_O_2_/min into the medium (Fig. 4) represents an intracellular production rate of 4.8 mM/min, or 80 μM/s. We make the assumption that the relationships between OD_600_ and cell volume are similar between *E. coli* and *B. thetaiotaomicron*; this notion is supported by the fact that we recover similar amounts of protein per OD unit.

The production of H_2_O_2_ was also measured during the respiration of inverted vesicles that had been prepared from *E. coli* Hpx^−^ or *B. thetaiotaomicron* Hpx^−^ cells. The cells had been grown in anaerobic minimal glucose media. Vesicles were added to 20 ml aerobic potassium phosphate buffer (50 mM [pH 7.8]) containing 0.4 mM succinate or 40 μM NADH. Superoxide dismutase (100 U/ml) was included in the reaction mixture. Where indicated, 3 mM malonate was used as an inhibitor of fumarate reductase. The reaction mixtures were shaken at 37°C, samples were removed at intervals, and their H_2_O_2_ content was measured by the Amplex Red-HRP method (64).

### Measurements of O_2_^−^ formation and ferricyanide and plumbagin reduction by inverted membrane vesicles.

Superoxide formation by inverted vesicles was measured in 1 ml KP_i_ buffer (pH 7.8) with succinate or NADH as the electron donor. The reduction of cytochrome *c* was monitored at 550 nm (ε = 21.0 mM^−1^ cm^−1^), in the presence and absence of SOD (27). The SOD-resistant activity represents direct reduction of cytochrome *c* by membrane enzymes, and this rate was subtracted from the no-SOD rate to derive the O_2_^−^ rate. Where indicated, 3 mM KCN was included to block possible electron flux from fumarate reductase through the cytochrome oxidase. Plumbagin reduction leads to immediate superoxide formation, so cytochrome *c* reduction was again monitored, in the presence of 3.2 mM succinate, 3 mM KCN, and 0.4 mM plumbagin (27). Ferricyanide reduction was monitored in the presence of respiratory substrate plus 0.2 mM potassium ferricyanide and 3 mM KCN; the reduction of ferricyanide was tracked by the disappearance of its absorbance at 420 nM (1 mM^−1^ cm^−1^).

SOD activities were measured by the xanthine oxidase-cytochrome *c* method (65).
