# Supplementary figures and images for: The Fumarate Reductase of Bacteroides thetaiotaomicron, unlike That of Escherichia coli, Is Configured so that It Does Not Generate Reactive Oxygen Species
Source: mBio. 2017 Jan 3;8(1):e01873-16. doi: 10.1128/mBio.01873-16 (PMC5210497; doi:10.1128/mBio.01873-16)

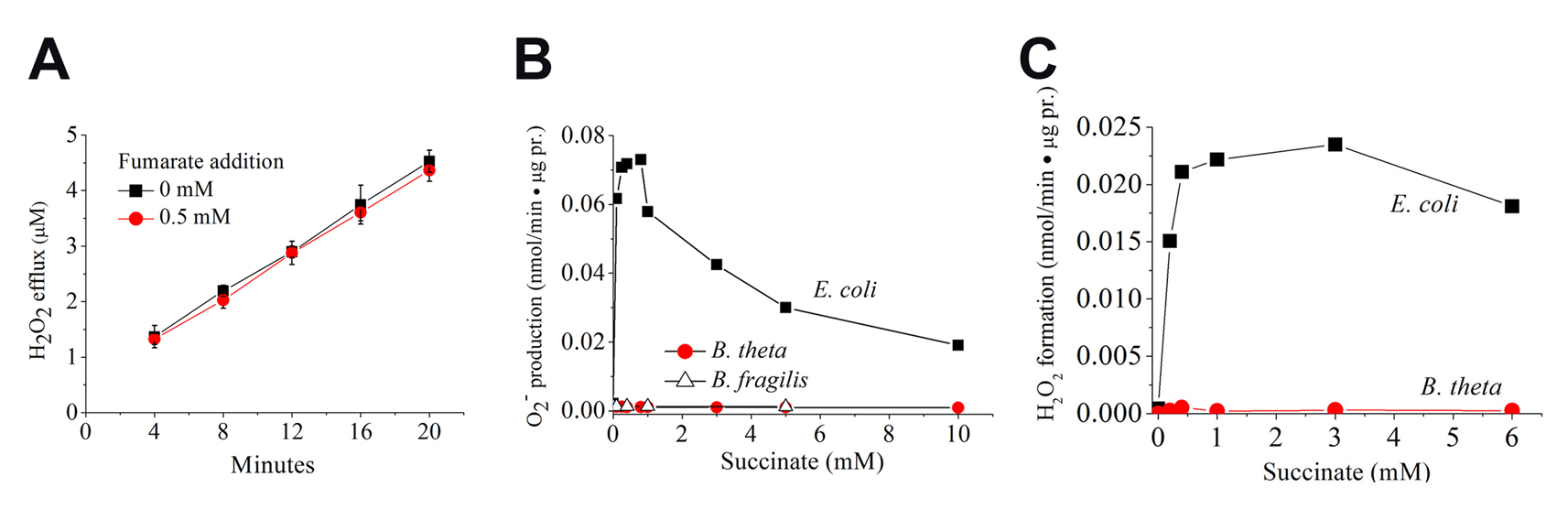

Supplement: Figure S1 [file mbo006163104sf1.tif]

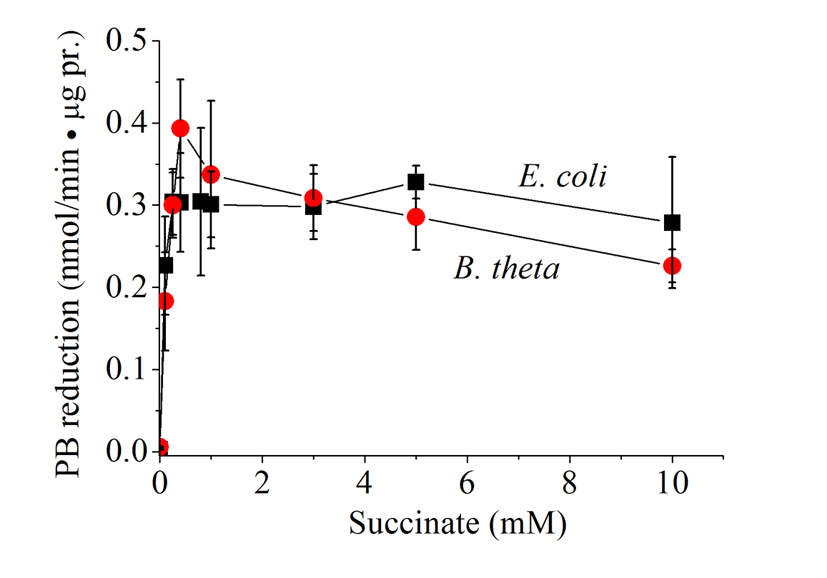

Supplement: Figure S2 [file mbo006163104sf2.tif]

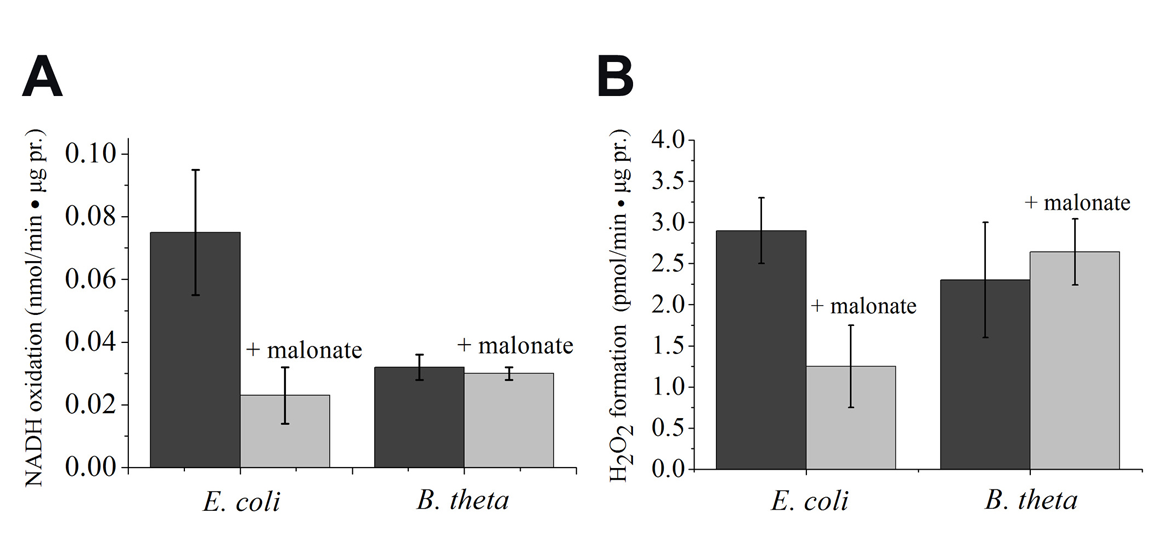

Supplement: Figure S3 [file mbo006163104sf3.tif]

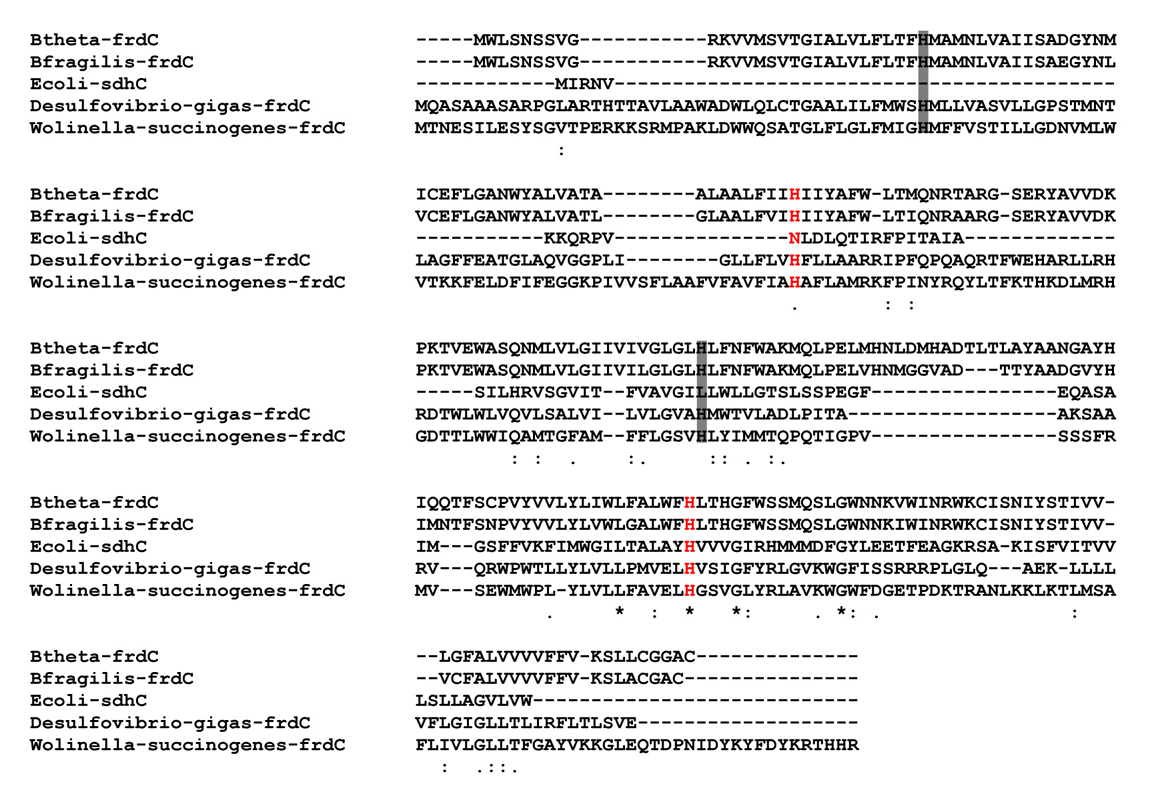

Supplement: Figure S4 [file mbo006163104sf4.tif]

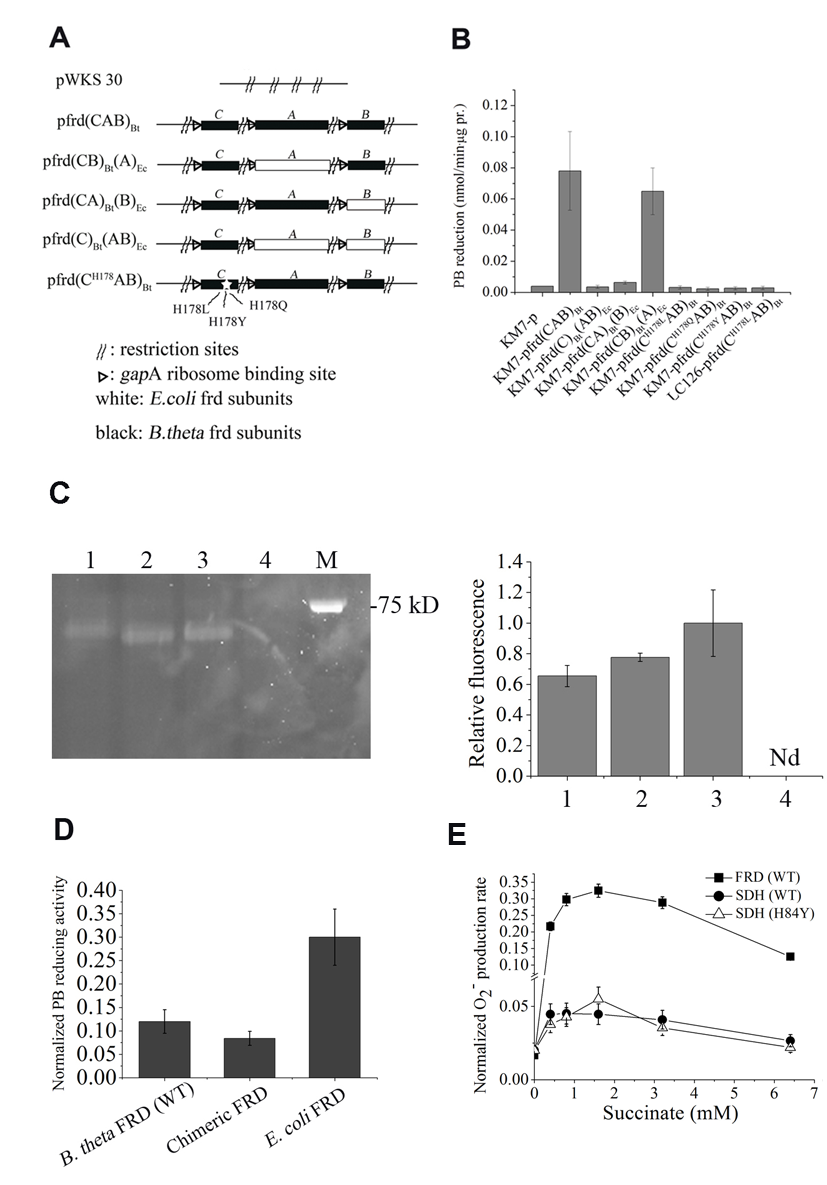

Supplement: Figure S5 [file mbo006163104sf5.tif]

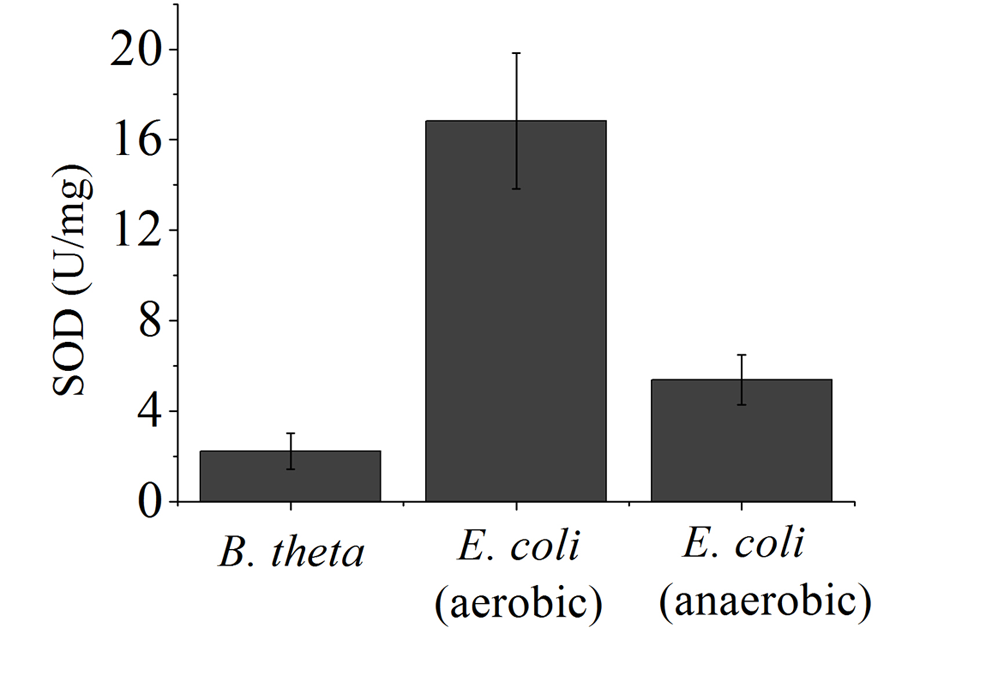

Supplement: Figure S6 [file mbo006163104sf6.tif]
