# Supplementary material for: The Fumarate Reductase of Bacteroides thetaiotaomicron, unlike That of Escherichia coli, Is Configured so that It Does Not Generate Reactive Oxygen Species
Source: mBio. 2017 Jan 3;8(1):e01873-16. doi: 10.1128/mBio.01873-16 (PMC5210497; doi:10.1128/mBio.01873-16)
Supplement: Table S1 [file mbo006163104st1.doc]

**Table S1. Fumarate reductase is the source of succinate-dependent ROS formation by *E. coli*** membrane vesicles.

|  | Rate (nmol/min • μg protein) | |
| --- | --- | --- |
| Cell membrane | Succinate:plumbagin reducing activity | Succinate:O_2_^-^ production |
| WT | 0.309 ±0.045 | 0.089±0.017 |
| △*sdh* | 0.258±0.050 | 0.095±0.012 |
| △*frd* | 0.009±0.004 | 0.007±0.005 |

Inverted vesicles were prepared from cells grown in anoxic minimal A glucose medium. The reactions contained 0.4 mM succinate and 3 mM KCN.
