# Supplementary material for: The Fumarate Reductase of Bacteroides thetaiotaomicron, unlike That of Escherichia coli, Is Configured so that It Does Not Generate Reactive Oxygen Species
Source: mBio. 2017 Jan 3;8(1):e01873-16. doi: 10.1128/mBio.01873-16 (PMC5210497; doi:10.1128/mBio.01873-16)
Supplement: Table S2 [file mbo006163104st2.doc]

**Table S2. O_2_ consumption by aerated *B. thetaiotaomicron*.**

| Strain | Respiration rate (μM O_2_/min • OD) |
| --- | --- |
| WT  △*cydAB* | 5.1±1.0  4.9±0.9 |
| △*roo* | 2.8±0.7 |

Cells were grown to exponential phase in anoxic BHIS medium, harvested, and transferred to oxic 37°C PBS containing glucose. O_2_ consumption was measured with a Clark oxygen electrode.
