# Supplementary material for: The Fumarate Reductase of Bacteroides thetaiotaomicron, unlike That of Escherichia coli, Is Configured so that It Does Not Generate Reactive Oxygen Species
Source: mBio. 2017 Jan 3;8(1):e01873-16. doi: 10.1128/mBio.01873-16 (PMC5210497; doi:10.1128/mBio.01873-16)
Supplement: Table S3 [file mbo006163104st3.doc]

**Table S3. H_2_O_2_** generation is diminished in rubredoxin-deficient strains.

| *B. thetaiotaomicron* strain | H_2_O_2_ generation (nM/min) |
| --- | --- |
| Hpx^-^ | 187.8 ±2.3 |
| Hpx^-^ △*rd* | 141.5±1.4 |
| Hpx^-^ △*nror* | 192.9±14.5 |
| Hpx^-^ △*roo* | 197.3±11.1 |
| Hpx^-^ △*rd* △*nror* | 153.2±10.7 |
| Hpx^-^ △*rd* △*nror* △*roo* | 160.6±8.4 |
