# Supplementary material for: The Fumarate Reductase of Bacteroides thetaiotaomicron, unlike That of Escherichia coli, Is Configured so that It Does Not Generate Reactive Oxygen Species
Source: mBio. 2017 Jan 3;8(1):e01873-16. doi: 10.1128/mBio.01873-16 (PMC5210497; doi:10.1128/mBio.01873-16)
Supplement: Table S4 [file mbo006163104st4.doc]

**Table S4. Strains and plasmids**

| **Strain** | | **Genotype** | **Source** |
| --- | --- | --- | --- |
| ***E. coli*** | MG1655 | F^-^ wild-type | *E. coli* Genetic Stock Center |
|  | Hpx^-^ (LC106) | MG1655 *ΔahpCF’ kan::’ahpF Δ(katG17::Tn10)1 Δ(katE12::Tn10)1* | Seaver and Imlay, 2004 |
|  | LC126 | LC106, *Δ(frdABCD)8…zjd::Tn10* | Seaver and Imlay, 2004 |
|  | KM7 | *Δ(frdABCD)8…zjd::Tn10 sdhC4*::*kan* | Lab collection |
|  | KM8 | *ubiA420 menA401 sdhC4*::*kan Δ(frdABCD)8…zjd::Tn10* | Lab collection |
|  | pH3 | plasmid encoding *E. coli* *frdABCD* | Blaut *et al.*, 1989 |
|  | pWKS30 | Empty vector, Amp^r^ | Wang and Kushner, 1991 |
|  | pfrd(CAB)_Bt_ | pWKS30 containing *B. thetaiotaomicron frdCAB*, Amp^r^ | This study |
|  | pfrd(C)_Bt_(AB)_Ec_ | pWKS30 containing *B. thetaiotaomicron frdC* plus *E. coli frdAB*, Amp^r^ | This study |
|  | pfrd(CA)_Bt_(B)_Ec_ | pWKS30 containing *B. thetaiotaomicron frdCA* plus *E. coli frdB*, Amp^r^ | This study |
|  | pfrd(CB)_Bt_(A)_Ec_ | pWKS30 containing *B. thetaiotaomicron frdC* plus *E. coli frdA* plus *B. thetaiotaomicron frdB*, Amp^r^ | This study |
|  | pfrd(C^H178L^AB)_Bt_ | pWKS30 containing *B. thetaiotaomicron frdC^H178L^AB*, Amp^r^ | This study |
|  | pfrd(C^H178Q^AB)_Bt_ | pWKS30 containing *B. thetaiotaomicron frdC^H178Q^AB*, Amp^r^ | This study |
|  | pfrd(C^H178Y^AB)_Bt_ | pWKS30 containing *B. thetaiotaomicron frdC^H178Y^AB*, Amp^r^ | This study |
|  | pFAS | PFRD *sdhC*(ATG)*DAB* Amp^r^ | Maklashina *et al*., 1998 |
|  | pFAS-H84Y | PFRD *sdhC*(ATG) ^H84Y^*DAB* Amp^r^ | Tran *et al*., 2007 |
| ***B. thetaiotaomicron*** | BT5482 *Δtdk* | Wild-type strain with *tdk* deletion; FUdR^R^ | Koropatkin *et al*., 2008 |
|  | Hpx^-^ (SM135) | BT5482 *Δtdk Δ(katE)1 Δ(ahpC)1 Δ(rbr)1 Δ(rbr2)1* | Mishra and Imlay, 2013 |
|  | SM021 | BT5482 *Δtdk Δ(oxyR)1* | Mishra and Imlay, 2013 |
|  | SM147 | BT5482 *Δtdk Δ(roo)1* | Lab collection |
|  | SM051 | BT5482 *Δtdk Δ(cydAB)1* | Lab collection |
|  | LZ01 | BT5482 *Δtdk Δ(sodA)1* (BT_0655) | This study |
|  | LZ53 | SM135, *Δ(rd)1* | This study |
|  | LZ55 | SM135, *Δ(nror)1* | This study |
|  | LZ67 | SM135, *Δ(roo)1* | This study |
|  | LZ61 | SM135, *Δ(rd)1 Δ(nror)1* | This study |
|  | LZ63 | SM135, *Δ(rd)1 Δ(nror)1 Δ(roo)1* | This study |
| ***B. fragilis*** | IB101 | 638R clinical isolate, Rif^r^ | Privitera *et al.*, 1979 |

**Strain sources.**

Blaut, M., Whittaker, K., Valdorvinos, A., Ackrell, B.A.C., Gunsalus, R.P., and Cecchini, G. (1989) Fumarate reductase mutants of *Escherichia coli* that lack covalently bound flavin. *J. Biol. Chem.* 264: 13599-13604.

Koropatkin, N.M., Martins, E.C., Gordon, J.I., and Smith, T.J. (2008) Starch catabolism by a prominent human gut symbiont is directed by the recognition of amylose helices. *Structure***16**: 1105-1115.

[Maklashina E](http://www.ncbi.nlm.nih.gov/pubmed/?term=Maklashina E%5BAuthor%5D&cauthor=true&cauthor_uid=12351213)., Berthold D.A. and [Cecchini G](http://www.ncbi.nlm.nih.gov/pubmed/?term=Cecchini G%5BAuthor%5D&cauthor=true&cauthor_uid=12351213). (1998) Anaerobic expression of *Escherichia coli* succinate dehydrogenase: functional replacement of fumarate reductase in the respiratory chain during anaerobic growth. *J. Bacteriol.* **180**:5989-5996.

Mishra S. and Imlay J.A. (2013) An anaerobic bacterium, *Bacteroides thetaiotaomicron*, uses a consortium of enzymes to scavenge hydrogen peroxide. *Mol. Microbiol.* **90**: 1356–1371.

Privitera, G., Dublanchet, A., and Sebald, M. (1979) Transfer of multiple antibiotic resistance between subspecies of *Bacteroides fragilis*. *J. Infect. Dis.* **139:**97-101.

Seaver, L. C., and Imlay, J.A. (2004) Are respiratory enzymes the primary sources of intracellular hydrogen peroxide? *J. Biol. Chem.* **279:** 48742-48750.

[Tran Q.M](http://www.ncbi.nlm.nih.gov/pubmed/?term=Tran QM%5BAuthor%5D&cauthor=true&cauthor_uid=17989224)., [Rothery R.A](http://www.ncbi.nlm.nih.gov/pubmed/?term=Rothery RA%5BAuthor%5D&cauthor=true&cauthor_uid=17989224)., [Maklashina E](http://www.ncbi.nlm.nih.gov/pubmed/?term=Maklashina E%5BAuthor%5D&cauthor=true&cauthor_uid=17989224)., [Cecchini G](http://www.ncbi.nlm.nih.gov/pubmed/?term=Cecchini G%5BAuthor%5D&cauthor=true&cauthor_uid=17989224)., [Weiner J.H](http://www.ncbi.nlm.nih.gov/pubmed/?term=Weiner JH%5BAuthor%5D&cauthor=true&cauthor_uid=17989224).(2007) *Escherichia coli* succinate dehydrogenase variant lacking the heme b*.* [*Proc. Natl. Acad. Sci. USA.*](http://www.ncbi.nlm.nih.gov/pubmed/?term=Tran+Quang+M.+et+al.%2C+2007) **104**:18007-12.

Wang, R.F., and Kushner, S.R. (1991) Construction of versatile low-copy-number vectors for cloning, sequencing and gene expression in *Escherichia coli*. *Gene* **100:** 195-199.
